# Non-Invasive Differentiation of M1 and M2 Activation in Macrophages Using Hyperpolarized ^13^C MRS of Pyruvate and DHA at 1.47 Tesla

**DOI:** 10.3390/metabo11070410

**Published:** 2021-06-22

**Authors:** Kai Qiao, Lydia M. Le Page, Myriam M. Chaumeil

**Affiliations:** 1 Department of Physical Therapy and Rehabilitation Science, University of California, San Francisco, CA 94143, USA; kai.tiong@ucsf.edu (K.Q.); lydia.lepage@ucsf.edu (L.M.L.P.); 2 Department of Radiology and Biomedical Imaging, University of California, San Francisco, CA 94143, USA

**Keywords:** Hyperpolarized ^13^C, MR spectroscopy, metabolism, macrophage activation, inflammation, innate immune response

## Abstract

Macrophage activation, first generalized to the M1/M2 dichotomy, is a complex and central process of the innate immune response. Simply, M1 describes the classical proinflammatory activation, leading to tissue damage, and M2 the alternative activation promoting tissue repair. Given the central role of macrophages in multiple diseases, the ability to noninvasively differentiate between M1 and M2 activation states would be highly valuable for monitoring disease progression and therapeutic responses. Since M1/M2 activation patterns are associated with differential metabolic reprogramming, we hypothesized that hyperpolarized ^13^C magnetic resonance spectroscopy (HP ^13^C MRS), an innovative metabolic imaging approach, could distinguish between macrophage activation states noninvasively. The metabolic conversions of HP [1-^13^C]pyruvate to HP [1-^13^C]lactate, and HP [1-^13^C]dehydroascorbic acid to HP [1-^13^C]ascorbic acid were monitored in live M1 and M2 activated J774a.1 macrophages noninvasively by HP ^13^C MRS on a 1.47 Tesla NMR system. Our results show that both metabolic conversions were significantly increased in M1 macrophages compared to M2 and nonactivated cells. Biochemical assays and high resolution ^1^H MRS were also performed to investigate the underlying changes in enzymatic activities and metabolite levels linked to M1/M2 activation. Altogether, our results demonstrate the potential of HP ^13^C MRS for monitoring macrophage activation states noninvasively.

## 1. Introduction

Nearly ubiquitous throughout the body, macrophages are a critical component in maintaining our health and wellbeing, playing a central role in the innate immune response, tissue homeostasis, and facilitation of crosstalk with neighboring cell types [1,2,3]. Macrophages have typically been described as having two activation states, which have each been observed to play significant roles in various pathologies, such as autoimmune disorders, obesity, and cancer malignancy [4,5,6]. Although there is still much to be understood, these activation states were first generalized to the M1 and M2 dichotomy [7], and are associated with cell-wide changes, including modulations of signaling pathways and reprogramming of cellular metabolism [8]. Briefly, M1 describes the classical proinflammatory activation response, leading to subsequent tissue damage, whereas M2 activation is associated with upregulation of anti-inflammatory pathways promoting tissue repair. From an energetic metabolism perspective, M1 macrophages have been shown to increase anaerobic glycolysis, while M2 use primarily aerobic oxidative phosphorylation for energy generation [9]. Reactive oxygen species (ROS) have also been reported to increase significantly in M1-activated macrophages, including in J774a.1 cells, and are often used as a marker of this activation state [10,11,12,13,14,15,16]. The activity of the arginase-1 enzyme isoform, meanwhile, has been shown to significantly increase specifically in M2-activated macrophages, serving as a robust marker of M2 activation [13,16,17,18,19,20,21].

Thus far, M1 and M2 differentiation has been largely determined using such markers by histological, biochemical, and genotypic studies conducted on samples collected invasively from the bloodstream or diseased tissues [22]. To date, no in vivo imaging method enables differentiation between M1 and M2 activation states. Given their broad and critical roles, such an approach could be very useful in improving our understanding of the role of macrophages in underlying diseases, and would also allow in vivo monitoring of responses to immunomodulatory treatments targeting macrophage activation in situ.

An investigative tool for assessment of in vivo metabolism exists in the form of ^13^C magnetic resonance spectroscopy (MRS) combined with dissolution dynamic nuclear polarization (dDNP) of ^13^C-labeled probes [23], so-called hyperpolarized (HP) ^13^C MRS. This technique has been used to noninvasively monitor metabolic impairment in multiple preclinical models, including cancer, neuroinflammation, and cardiomyopathy [24,25,26]. Importantly, the use of this technology is now expanding into the clinic, with ongoing clinical trials on patients with brain tumor, traumatic brain injury, and prostate cancer, among others [27,28,29]. Depending on the choice of ^13^C-labeled substrate, different metabolic pathways can be targeted and imaged noninvasively [30,31]. To date, two studies have used HP ^13^C MRS to look at activation states of macrophages. One study described the use of HP [6-^13^C]arginine to detect arginase activity in M2-like primary mouse myeloid-derived suppressor cells (MDSCs) [32]. The authors showed that metabolism of HP [6-^13^C]arginine to HP ^13^C urea was significantly increased in MDSCs compared to control bone marrow cells, in line with increased arginase activity linked to M2 activation. Two other studies on macrophage cells (J774a.1 [33] and RAW264.7 [34]) using HP [1-^13^C]pyruvate showed that, after M1 activation using the toxin lipopolysaccharides (LPS), the conversion of HP [1-^13^C]pyruvate into [1-^13^C]lactate, catalyzed by the lactate dehydrogenase (LDH) enzyme, was significantly increased compared to nontreated macrophages. This result was linked to, among other events, increased LDH activity and gene transcription [33]. Furthermore, blockade of glycolysis with 2-deoxyglucose in activated cells normalized lactate label flux rates and markedly inhibited the production of key proinflammatory cytokines [34]. However, to date, no study has directly compared M1 vs. M2 activation states in macrophages using HP ^13^C MRS, and no other HP ^13^C probe besides HP [1-^13^C]pyruvate has been tested in macrophage cell lines.

**Figure 1:**
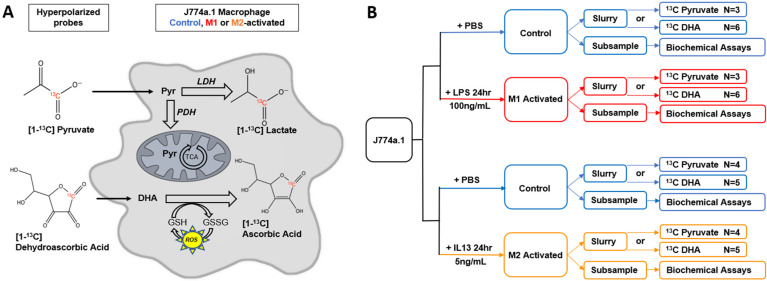
Overall design. (**A**) Schematic of hyperpolarized ^13^C probes and their metabolic fates in J774a.1 macrophages. LDH = lactate dehydrogenase; PDH = pyruvate dehydrogenase; TCA = tricarboxylic acid cycle; GSH = glutathione; GSSG = glutathione disulfide; ROS = reactive oxygen species. (**B**) Overall experimental design of the study. Activation was achieved with either lipopolysaccharide (LPS, M1 activation) or interleukin-13 (IL-13, M2 activation). Control and M1/M2 activated samples were paired for HP studies and for spectrophotometric assays. N = number of repeats.

In this comprehensive study, we applied HP ^13^C MRS to paired M1 vs. control, and paired M2 vs. control macrophages. Two metabolic conversions were evaluated: (1) the conversion of HP [1-^13^C]pyruvate into HP [1-^13^C]lactate, and (2) the conversion of HP [1-^13^C]dehydroascorbic acid (DHA) into HP [1-^13^C]ascorbic acid (AA) (Figure 1). The first reaction is the final step of anaerobic glycolysis, while the second reaction is modulated by ROS levels. Both reactions have previously been successfully imaged in vivo [35,36]. In our study, we show that, for both probes, HP product-to-substrate ratios were observed to be significantly increased in M1 macrophages compared to M2, while control and M2 macrophages presented similar metabolic ratios. Underlying mechanisms were investigated, and spectrophotometric assays showed that changes in HP lactate-to-pyruvate ratios were associated with changes in LDH and pyruvate dehydrogenase (PDH) enzymatic activities, while changes in HP AA-to-DHA ratio paralleled changes in ROS levels.

Our study therefore shows that noninvasive differentiation of M1 vs. M2 activation in macrophages can be achieved using ^13^C MRS of HP [1-^13^C]pyruvate and DHA at the clinically relevant field strength of 1.47 Tesla. Upon translation to in vivo models, this method could prove useful to evaluate the immune response and monitor the effect of immunomodulatory treatments.

## 2. Results

### 2.1. M1 and M2 Activation of J774a.1 Macrophages

First, ROS and Arginase assays were performed to confirm differential M1 and M2 activation of J774a.1 macrophages, which was performed using previously established protocols ([33] for M1 and [37] for M2). Arginase enzyme activity was unchanged with M1 activation by LPS (*p* = 0.849), while a significant 57-fold increase in arginase activity was seen in M2 macrophages activated by interleukin-13 (IL-13) (*p* < 0.0001, N = 3, 38.255 U/L/10^5^ cells in M2-activated vs. 0.669 U/L/10^5^ cells for control), as previously reported [37] (Supplementary Figure S1A). On the other hand, M1-activated macrophages exhibited a 2.3-fold increase in ROS compared to control (*p* = 0.0009, N = 6), whereas no significant increase in ROS was detected in M2-activated macrophages (*p* = 0.7380, N = 3) (Supplementary Figure S1B). The ROS increase in M1-activated macrophages is in agreement with previous reports [38]. The Bradford assay showed no differences in total protein concentration per cell between groups (Supplementary Figure S1C).

### 2.2. HP [1-^13^C]lactate Production Is Differentially Increased by M1/M2 Macrophage Activation

**Figure 2:**
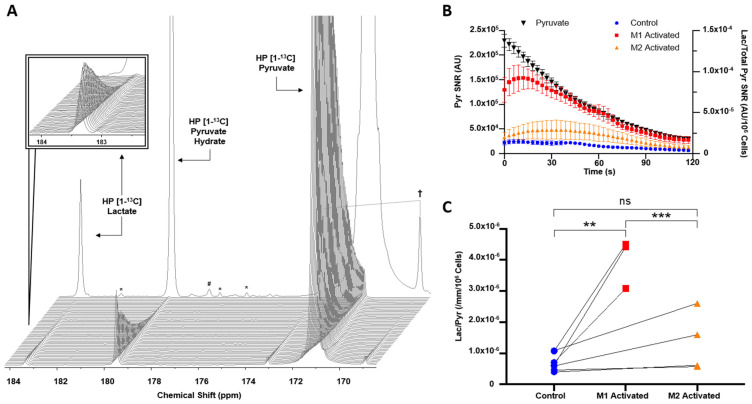
HP [1-^13^C]lactate production is differentially increased by M1/M2 macrophage activation. (**A**) Dynamic spectra acquired at 1.47 Tesla post HP [1-^13^C]pyruvate injection into M1-activated J774a.1 macrophages, and corresponding sum spectra over 100 TRs in background. Insert shows HP [1-^13^C]lactate dynamics. († symmetric splitting of pyruvate due to 1.1% ^13^C natural abundance in C2 position; # alanine peak; * contaminants). (**B**) Signal to noise ratio (SNR) of HP [1-^13^C]pyruvate decay from all injections (black, left *y*-axis) and HP [1-^13^C]lactate production (right *y*-axis) in M1-activated (red), M2-activated (orange), and control cells (blue) over 120 s. (**C**) HP lactate-to-pyruvate ratios measured in controls (blue) and their activation pairs (M1-activated red, M2-activated orange) (*Non Significant NS*: *p* = 0.1866, ** *p* < 0.01, *** *p* < 0.001).

The T1 of HP [1-^13^C]pyruvate was calculated to be 52 s at 1.47 Tesla. Upon injection of HP [1-^13^C]pyruvate into activated and control macrophages, HP [1-^13^C]lactate production could be detected at 183.3 ppm, with the HP [1-^13^C]pyruvate resonance visible at 171.1 ppm (Figure 2A). Figure 2B shows the mean HP [1-^13^C]lactate signal for control, M1-activated, and M2-activated datasets over the first 40 TRs normalized to cell number. The buildup of HP [1-^13^C]lactate can be observed, demonstrating in situ metabolism. Quantification of the total sum spectra yielded a non-normalized HP [1-^13^C]pyruvate SNR of 6.06 ± 1.76 × 10^5^, 5.33 ± 2.33 × 10^5^, and 5.89 ± 1.25 × 10^5^ for control, M1-activated, and M2-activated, respectively, while the SNR of HP [1-^13^C]lactate for control, M1, and M2 was 1.27 ± 0.68 × 10^3^, 2.28 ± 0.35 × 10^3^, and 2.44 ± 0.73 × 10^3^, respectively. When normalizing to cell number and volume, in comparison to control cells, HP lactate-to-pyruvate ratio was significantly increased by 467 ± 49% with M1 activation (*p* = 0.0010, 4.00 ± 0.27 × 10^−6^ for M1-activated cells vs. 7.06 ± 0.39 × 10^−7^ for control cells). In M2 macrophages, a 91 ± 36% increase was observed compared to controls, but it did not reach significance (*p* = 0.1866, 1.35 ± 0.24 × 10^−6^ for M2-activated cells) (Figure 2C). When comparing activation states, HP lactate-to-pyruvate ratio was significantly higher by 197 ± 57% in M1-activated cells as compared to M2-activated cells (*p* = 0.0008).

### 2.3. HP [1-^13^C]DHA Conversion to HP [1-^13^C]AA Is Increased with M1 Activation

**Figure 3:**
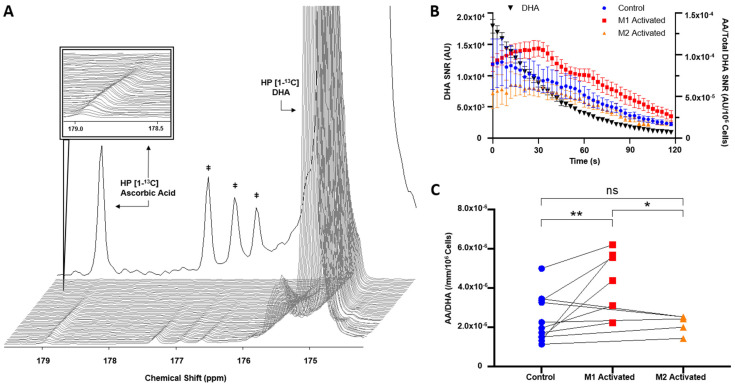
HP [1-^13^C]AA production is increased with M1 activation. (**A**) Dynamic spectra acquired at 1.47 Tesla post HP [1-^13^C]DHA injection into M1-activated J774a.1 macrophages, and sum spectra over 100 TRs in background (‡ contaminants). (**B**) Signal-to-noise ratio (SNR) of HP [1-^13^C]DHA decay from all injections (black, left *y*-axis) and HP [1-^13^C]AA production (right *y*-axis) in M1-activated (red), M2-activated (orange), and control cells (blue) over 120 s. (**C**) HP AA-to-DHA ratios measured in controls (blue) and their activation pairs (M1-activated red, M2-activated orange) (*NS*: *p* = 0.9581, * *p* < 0.05, ** *p* < 0.01).

The T1 of HP [1-^13^C]DHA was calculated to be 46 s at 1.47 Tesla. Following injection of HP [1-^13^C]DHA, HP [1-^13^C]AA production could be detected at 178.8 ppm in control and activated macrophages, while the resonance of the substrate HP [1-^13^C]DHA was detected at 175 ppm (Figure 3A). The time courses of HP [1-^13^C]DHA and HP [1-^13^C]AA are shown in Figure 3B for a control, an M1, and an M2-activated dataset. HP [1-^13^C]AA signal gradually builds up as the signal from HP [1-^13^C]DHA decays, denoting in situ conversion. Quantification of the total sum spectra yielded a non-normalized HP [1-^13^C]DHA SNR of 2.23 ± 0.77 × 10^4^, 3.16 ± 0.71 × 10^4^, and 2.51 ± 1.03 × 10^4^ for control, M1-activated, and M2-activated, respectively, while the SNR of HP [1-^13^C]AA for control, M1, and M2 was 142 ± 58, 217 ± 67, and 144 ± 67, respectively. When normalizing to cell number and volume, HP AA-to-DHA ratio was significantly increased by 88 ± 14% in M1 compared to control (*p* = 0.0034, 4.53 ± 0.26 × 10^−6^ in M1-activated vs. 2.41 ± 0.11 × 10^−6^ in control), while no significant differences were observed in M2 macrophages vs. control (*p* = 0.9581, 2.19 ± 0.10 × 10^−6^ in M2-activated) (Figure 3C). When comparing activation states, HP AA-to-DHA ratio was significantly higher by 107 ± 15% in M1 cells as compared to M2-activated cells (*p* = 0.0122).

### 2.4. LDH Activity Is Increased in Both M1- and M2-Activated Macrophages, While PDH Activity Is Decreased in M1 and Increased in M2 Activation

**Figure 4:**
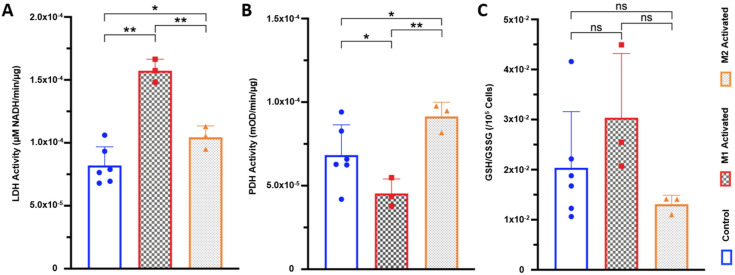
LDH and PDH activities are differentially modulated with M1/M2 activation (**A**) Lactate dehydrogenase (LDH) enzyme activity for control (blue), M1-activated (red), and M2-activated (orange) macrophages (* *p* < 0.05, ** *p* < 0.01, normalized to protein concentration). (**B**) Pyruvate dehydrogenase (PDH) enzyme activity measured by spectrophotometry for control (blue), M1-activated (red), and M2-activated (orange) macrophages (* *p* < 0.05, ** *p* < 0.01, normalized to protein concentration). (**C**) Reduced glutathione (GSH) over oxidized glutathione (glutathione disulfide, GSSH) GSH/GSSG ratio per 105 cells for control (blue), M1-activated (red), and M2-activated (orange) macrophages (*NS* control vs. M1: *p* = 0.1265; *NS* control vs. M2: *p* = 0.8326; *NS* M1 vs. M2: *p* = 0.1083).

Interestingly, LDH enzyme activity was significantly increased by 92 ± 6% in M1-activated macrophages (*p* = 0.0020, 1.57 × 10^−4^ µM NADH/min/µg for M1-activated vs. 8.20 × 10^−5^ µM NADH/min/µg for control), and by 27 ± 6% in M2-activated macrophages (*p* = 0.0300, 1.04 × 10^−4^ µM NADH/min/µg) (Figure 4A). In contrast, PDH activity was significantly decreased by 34 ± 5% in M1-activated macrophages (9.08 × 10^−4^ mOD/min/µg) compared to control (1.37 × 10^−3^ mOD/min/µg, *p* = 0.0297). Meanwhile, M2-activated macrophages showed a significant 34 ± 7% increase in PDH activity (1.83 × 10^−3^ mOD/min/µg) compared to control (*p* = 0.0290) (Figure 4B). Spectrophotometric assay of glutathione (GSH) to glutathione disulfide (GSSG) GSH/GSSG ratios with subsequent normalization to 10^5^ cells showed no significant differences between groups (Figure 4C).

### 2.5. High Resolution ^1^H NMR Detects Significant Differences in Extracted Metabolites between Control, M1, and M2 Macrophages

**Figure 5:**
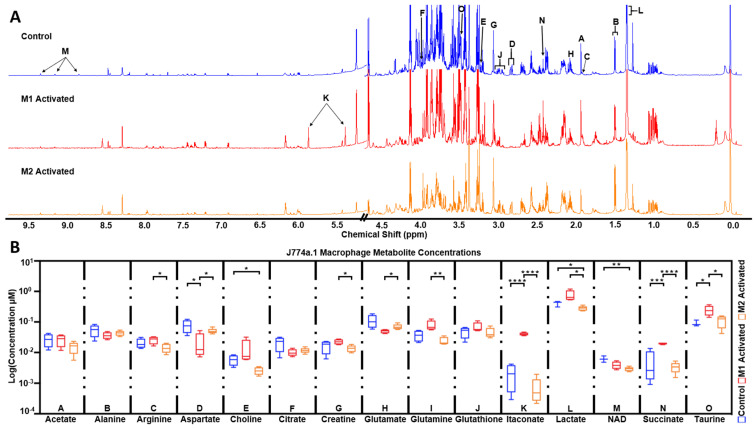
High resolution ^1^H NMR detects significant differences in extracted metabolites between control, M1, and M2 macrophages. (**A**) Representative high resolution ^1^H NMR 1D NOESY spectra of control (blue), M1-activated (red), and M2-activated (orange) J774a.1 macrophage extracts (water region omitted; normalized to cell number with axes scaled identically). (**B**) Metabolite concentrations between control (blue; N = 4), M1-activated (red; N = 5), and M2-activated (orange; N = 5) determined from ^1^H NMR data. Data reported in log for purpose of presentation, with statistics performed on metabolite concentration (µM) normalized to cell number and TSP. Significance from two-way ANOVA shown between relevant groups with * *p* < 0.05, ** *p* < 0.01, *** *p* < 0.001, **** *p* < 0.0001.

^1^H NMR spectra of extracted metabolites exhibited well-resolved peaks at 800 MHz (Figure 5A). Multiple metabolite concentrations were observed to be significantly altered between groups (Figure 5B), with many in agreeance with previous reports [33]. In M1-activated macrophages, significant increases in itaconate (*p* < 0.0001 vs. control, *p* < 0.0001 vs. M2), taurine (*p* < 0.05 vs. control, *p* < 0.05 vs. M2), and succinate (*p* < 0.001 vs. control, *p* < 0.0001 vs. M2) were measured, whereas a significant decrease in aspartate (*p* < 0.05 vs. control, *p* < 0.05 vs. M2) was detected. In M2 macrophages, significant decreases in NAD (*p* < 0.01 vs. control, *Non Significant (NS)* vs. M1), lactate (*p* < 0.05 vs. control, *p* < 0.05 vs. M1), and choline (*p* < 0.05 vs. control, *NS* vs. M1) were observed. Additional significant metabolite changes between M1- and M2-activated macrophages include glutamate (*p* < 0.05), creatine (*p* < 0.05), and arginine (*p* < 0.05). Metabolite concentrations for each activation group are detailed in Table 1.

## 3. Discussion

In this study, we used the established J774a.1 mouse macrophage cell line and applied M1 [33] and M2 [37] activation protocols described in previous reports. First, we confirmed that differential activation was successfully achieved, as shown by a significant increase of arginase activity in M2 macrophages [20] and an increased level of ROS in M1 macrophages [7]. Of note, we did not see a significant decrease in ROS levels in M2-activated macrophages compared to control, as others have reported [22], likely due to the small number of cells available for this assay. Protein concentrations per cell were unchanged with M1 or M2 activation, justifying the normalization of all HP and spectrophotometric values to number of cells throughout the study. The ^1^H MRS results further confirmed differential activation of macrophages. Highly significant increases in itaconate and succinate concentrations are seen in M1 macrophages compared to control, in line with previous reports [39], as itaconate is a well-known product of M1 polarization [40] and a potent inhibitor of succinate dehydrogenase, which leads to succinate accumulation [41]. Glutamine is higher in M1 compared to control, albeit not significantly, in line with a previous report utilizing the identical cell line and activation protocol [33], and also consistent with a study of LPS-induced M1 polarization utilizing a tenfold lower dosage (10 ng/mL) [42]. The increased lactate observed in M1 compared to control and M2 is also indicative of highly upregulated lactate production and Warburg-like effect that are specific to M1 activation [22]. In M2-activated macrophages, the observed decrease in arginine levels compared to M1 could be explained by increased arginine consumption through upregulated arginase activity, though, to date, the only metabolomics study of macrophage activation reports increased intracellular arginine concentration for both M1 and M2 [43]. It should be noted, however, that this study used primary human macrophages, which have been shown to respond differently than established cell lines, as well as much longer activation protocol of 72 h, which might explain the observed discrepancy [44]. A larger metabolomics study specific to activation patterns of murine macrophage cell lines would need to be pursued for more direct comparisons.

Here, we used an innovative approach allowing for noninvasive assessment of metabolism, namely HP ^13^C MRS, and evaluated its potential to differentiate between activation states in live macrophages. Our results show that significant differences in HP product-to-substrate ratios of both HP [1-^13^C]pyruvate and HP [1-^13^C]DHA can be observed in differentially activated murine macrophages. The sampling of cell slurries prior to each HP ^13^C MRS acquisition allowed for paired biochemical assays to be performed, thus enabling for strong biochemical validations. The methods used may be applied in other cell types, both from culture and primary populations.

The increased HP ^13^C lactate-to-pyruvate ratio observed in M1-activated macrophages is in agreeance with previous reports [33], and congruent with the expected highly increased glycolytic activity in proinflammatory macrophages [45]. This increased ratio is also associated with a significant increase in LDH activity in the M1 group detected in paired subsamples. In M2-activated macrophages, the HP ^13^C lactate-to-pyruvate ratio was not significantly different from control, although a trend towards significance was observed. This trend is in line with a smaller significant increase in LDH activity, which was detected by spectrophotometric assays that are likely more sensitive than HP ^13^C MRS. These results are also in agreement with previous reports showing that M2 polarized macrophages exhibit a more modest increase in glycolytic activity compared to nonactivated macrophages [46]. To further understand our HP results, it is important to look into another common pathway for HP [1-^13^C]pyruvate metabolism, through PDH. In M1-activated macrophages, our spectrophotometric results showing decreased PDH activity compared to controls are in line with previous reports showing significantly reduced PDH activity in M1 primary murine bone-marrow-derived macrophages [39]. Reduced PDH activity in M1 macrophages would lead to less HP [1-^13^C]pyruvate entering the Krebs cycle, shuttling this HP probe towards LDH and increased HP [1-^13^C]lactate production, in line with our HP results. In M2-activated macrophages, on the other hand, we observed a significant increase in PDH activity. This result is interesting, and reasonable given that expression of pyruvate dehydrogenase kinase (PDK), an inhibitor of PDH, is reduced in M2 polarization [47]. In that case, and contrary to M1, increased PDH activity may lead to more HP [1-^13^C]pyruvate being shuttled into the mitochondria, thus decreasing flux towards HP [1-^13^C]lactate production via LDH. This result provides an additional explanation for the fact that HP lactate–pyruvate ratio was not significantly increased in M2-activated macrophages, despite the increased LDH activity. It should be noted that HP [1-^13^C]pyruvate metabolized through PDH can generate HP [1-^13^C]bicarbonate via pyruvate decarboxylation, and measurements of HP [1-^13^C]bicarbonate have been used to determine PDH flux in previous studies [48,49]. Although the spectral bandwidth in this study was large enough to cover the resonance of HP [1-^13^C]bicarbonate at 163 ppm, the signal of this metabolite was not detected, likely due to its intrinsically low level in this cell type. Finally, in addition to enzymatic activities, the levels of steady-state lactate levels from ^1^H NMR might also contribute to the HP readouts through the well-documented pool-size effect [50,51,52]. The observed significant decrease in ^1^H lactate levels in M2 macrophages may also contribute to the reduced HP lactate levels in that group. In M1 macrophages, lactate levels follow an increasing trend (*p* = 0.128), which is also in line with the HP results. Multiple additional factors contributing to the HP readouts could be considered, including levels of membrane transporters (e.g., MCT1) or NAD cofactor availability. However, such measures could not be performed in this study due to the limited number of paired samples available.

Our results show that conversion of HP [1-^13^C]DHA to HP [1-^13^C]AA was increased in M1 macrophages, mirrored by increased ROS levels, but unexpectedly not by changes in GSH levels or GSH/GSSG ratio. It is well known that the GSH redox system is a mitigator of ROS [53], while also being coupled to the conversion of DHA into AA [54]: GSH is oxidized to GSSG by DHA, which, in turn, is converted to AA. Increasing ROS levels should theoretically deplete the available pool of GSH (and increase levels of GSSG), leaving less GSH available for the production of AA, and thus leading to a decreased HP AA-to-DHA ratio, as previously reported [36,55]. However, both GSH/GSSG ratios, as detected by spectrophotometric assay, and total GSH levels, as detected by ^1^H NMR, were not significantly different between M1 macrophages and control or M2. A previous study of the same cell line also showed that the total pool of GSH as detected by ^1^H NMR was not increased post-activation by LPS [33]. Other reports using biochemical methods also show no changes in total GSH levels with M1 activation, but do note elevated GSH/GSSG ratio [56]. Elevated GSH/GSSG ratio can explain an increase in AA production, and may be a compensatory effect unique to macrophages, as they endogenously manufacture ROS as part of the immune response. Our results show that a trend to an increase in M1-activated compared to control (*p* = 0.1265) and M2 (*p* = 0.1083) was seen, although it did not reach significance, possibly due to the limited numbers of available paired samples (N = 3) that were spread thinly across the numerous assays. Further work could be done with a larger sample size to confirm the increase in GSH/GSSG ratio in M1 macrophages. Importantly, conversion of HP [1-^13^C]DHA to HP [1-^13^C]AA can also be affected by the levels of the glucose membrane transporter 1 (GLUT1), which is a known facilitated transporter of DHA [57]. GLUT1 has been shown to be upregulated with M1 activation compared to quiescent [58,59], whereas its expression is comparable between control and M2-activated states [42,60]. These facts mirror our HP AA/DHA ratio results and could be another possible mechanistic explanation to our observed HP data. Due to the limited number of paired samples, however, membrane transporter expressions were not evaluated here.

The power of dynamic metabolic probing using HP ^13^C MR is compelling, as it allows the measuring of previously inaccessible metabolic reactions noninvasively. HP ^13^C metabolic imaging is a rapidly growing field, and is now being used in multiple clinical trials across the globe, targeting several organs, including the brain, heart, prostate, and kidney [28,29,61,62,63,64,65,66,67,68,69,70]. It is very likely that, given the constant improvements reported, both on the acquisition and processing sides, this methodology will soon approach feasibility for widespread clinical adoption. Currently, the most popular probe for hyperpolarization is HP [1-^13^C]pyruvate, with some of the best polarization characteristics [52], but other probes are continuously being investigated, opening up possibilities not explored before. While the clinical data reported so far are highly compelling, mechanistic studies are still critically needed to understand the relative contribution of each cell type to the detected HP signal, especially for cells as ubiquitous as macrophages, which are found in most diseases and most organs. Here, we performed the first study of live macrophages at a clinically relevant field strength, and compared both M1 and M2 activation patterns. Before, the only other study that used HP ^3^C MRS on activated macrophages was conducted at the high magnetic field strength of 11.7 Tesla, and employed M1 activation only [33]. We showed that, at clinical field strength, the spectral resolution was sufficient to enable measurements of metabolic fluxes in live cells. Further, we demonstrated that M1 and M2 macrophages have a different HP metabolic signature, with both HP [1-^13^C]pyruvate and HP [1-^13^C]DHA. It is important to note that incubation with LPS and IL-13 is only one way to induce M1 and M2 activation, respectively. Other cytokines, or cytokine cocktails, could be used to induce M1 and M2 activation states (e.g., IL-14 for M2 [71], or interferon-gamma (IFN-γ) for M1 [72]), and further studies would be needed to confirm that the HP results observed in this study hold across activation protocols, as well as in vivo in preclinical models. Further, potential changes in metabolism caused by the change in cellular environment from flask to NMR tube would require additional investigation. Nevertheless, this study further establishes HP metabolic imaging as an interesting tool to assess inflammation, and our results could help increase the understanding of the metabolic readouts observed in vivo in preclinical models and patients.

## 4. Materials and Methods

### 4.1. Cell Culture

J774a.1 mouse macrophages (ATCC, Manassas, VA, USA) were grown in Dulbecco’s modified Eagle’s media (DMEM) containing 10% fetal bovine serum and 5% penicillin/streptomycin (UCSF). M1 activation was achieved with 100 ng/mL LPS treatment for 24 h (*E. coli*; Sigma Aldrich), and M2 activation with 5 ng/mL of murine IL-13 for 24 h (Peprotech), as previously described [32,36]. A control group was established with a vehicle treatment of sterile PBS. All passage numbers used were ~4–14 to reduce the risk of genetic drift, and a mycoplasma testing kit (ATCC) confirmed the culture was contamination-free. Figure 1 presents a schematic of the full experimental design.

For HP ^13^C experiments, cells were studied as paired samples of either M1 or M2 activation and paired controls. The definition of paired samples were as follows: three T225 flasks were split into ten T225 flasks, five of which were activated towards either M1 or M2, and the other five with vehicle as control. The adherent macrophages were incubated with 0.04% EDTA in calcium and magnesium-free PBS solution (Cell Culture Facility, UCSF) for approximately 10 min, collected, and then centrifuged at 125× *g* for 5 min. The pellet was resuspended in 200 μL of fresh DMEM (no additives), and a 20 μL sample was taken and washed with PBS to be saved at −80 °C for paired spectrophotometric assays (for all assays except ROS). Another 20 μL sample was taken for cell counting (referred to as subsample in the rest of this manuscript), and the remaining slurry suspension (~20 million cells) was transferred to a 5 mm NMR tube. All injections of HP probes were done within 5 min of cell resuspension and transfer to the NMR tube.

### 4.2. MR Acquisitions

A total of 24 μL of [1-^13^C]pyruvate (15 M pyruvic acid (Sigma Aldrich, St Louis, MO, USA), 15 mM trityl radical (GE), 500 mM Gd-DOTA (Guerbet, Roissy, France)) or 25 μL of [1-^13^C]DHA (2.2 M dehydroascorbic acid (Sigma) prepared as previously described [36]) was polarized for 1 h on a Hypersense dDNP polarizer (Oxford Instruments), then dissolved in 4.5 mL or 3.5 mL buffer (pyruvate buffer: 80 mM NaOH, 40 mM Tris HCl, 3 mM EDTA in ddH_2_O; DHA buffer: 3 mM EDTA in ddH_2_O), to yield a final solution of 80 mM or 15.72 mM, respectively. Previous formulations of [1-^13^C]pyruvate and [1-^13^C]DHA polarized on the Hypersense polarizer achieved 13.9–27.5% and ~10% polarization, respectively [55,73]. Within 20 s of dissolution, approximately 400 μL of HP [1-^13^C]pyruvate (n = 3 control vs. M1-activated pairs, n = 4 control vs. M2-activated pairs) or [1-^13^C]DHA (DHA: n = 6 control vs. M1-activated pairs, n = 5 control vs. M2-activated pairs) was injected into a 5 mm NMR tube containing a ~20 million cell slurry in 200 μL DMEM. Hyperpolarized spectra were then acquired on a 1.47 T Oxford Pulsar NMR system using a 1D pulse acquire sequence with the following parameters: flip angle = 20°, repetition time (TR) = 3 s, pulse type = WALTZ-4, pulse length = 4 μs, and number of scans (NS) = 100, for a total acquisition of 5 min. Analysis was performed with Mestrenova (Mestrelab, Santiago de Compostela, Spain) software. Signal to noise ratio (SNR) for the injected substrates and detected products were calculated from the total summed spectra as area under curve divided by standard deviation of the noise. Contaminants were identified via blank experiments with hyperpolarized substrates injected into NMR tubes with media only. Spin-lattice relaxation time T1 of each HP probe was also calculated from such blank experiments. All HP data are represented as mean ± standard error of the mean (SEM), and are normalized to cell number and volume of injection.

### 4.3. Spectrophotometric Assays

ROS levels, arginase activity, LDH activity, PDH activity, and GSH levels were measured with spectrophotometry on paired samples collected from the same flasks as the ones used for HP experiments. ROS data of control (N = 6), M1-activated (N = 6), and M2-activated (N = 3) were reported as fold-change from control using a commercial intracellular ROS fluorescence assay kit (Abcam, Cambridge, UK), used according to manufacturer’s instructions). Arginase assay (Abcam) activity of control (N = 6), M1-activated (N = 3), and M2-activated (N = 3) was reported in units/Liter (U/L) and normalized to 10^5^ cells per well. An in-house LDH assay measuring the rate of NADH (Sigma) depletion for control (N = 6) and activated groups (N = 3 for both M1 and M2) was performed and reported as µM NADH/minute normalized to protein concentration quantified by Bradford assay (Thermofisher, Waltham, MA, USA). A PDH assay kit (Abcam, Cambridge, UK) was used to measure PDH enzyme activity between groups (N = 3 for each control, M1, and M2), and reported as optical density (milliOD)/minute with normalization to protein concentration. GSH and its oxidized form GSSG were measured with a commercial kit (Biovision, San Francisco, CA, USA). The total glutathione levels were first measured, then subtracted from measured levels of GSH to obtain GSSG values, with the subsequent GSH/GSSG ratio reported with normalization to 10^5^ cells per well. All spectrophotometric data are reported as mean ± standard deviation.

### 4.4. High Resolution ^1^H NMR of Extracted Cell Metabolites

Metabolites from M1-activated (N = 5), M2-activated (N = 5), and control (N = 4) J774a.1 cells were extracted using equal parts methanol–water–chloroform, as previously described [74]. Cold 4 °C saline (5 mL) was added directly to T75 flasks 2–3 times and removed to rinse, and −20 °C methanol (3 mL) was subsequently added. The adherent macrophages were scraped off into the methanol—this mixture was transferred to a clean tube. Equal parts −20 °C chloroform and 4 °C H_2_O were homogenously mixed, and the fractions separated by centrifuging at 125× *g* at 4 °C. The methanol fraction was collected, 0.65 mM trimethylsilylpropanoic acid (TSP) (Acros Organics) added, and the mixture lyophilized. The resultant extracts were reconstituted in 420 μL D_2_O, and the samples were scanned on an 800 MHz NMR system (Bruker, Billerica, MA, USA) with a 1D NOESY presaturation sequence with TR = 3.31 s, mixing time = 50 ms, and NS = 384. A subsequent fully relaxed scan was performed with a relaxation delay of 30 s to ensure a time of at least 5 times the longest T1 based on literature values [75]. This spectrum was used to generate correction factors that were then subsequently applied to all spectra to enable absolute quantification of metabolites. Spectral processing was performed with Mestrenova (Mestrelab, Santiago de Compostela, Spain), and a select group of metabolites of interest previously reported in macrophage studies [33] were fitted and quantified using Chenomx NMR Suite (Chenomx Inc, Edmonton, AB, Canada) with reference to the Human Metabolomics Database [76]. Concentrations of quantified metabolites were normalized to cell number and TSP reference, and reported as mean ± standard deviation.

### 4.5. Statistical Analyses

The sample sizes of HP experiments were determined using an 80% power calculation of preliminary paired data between activated and nonactivated groups. All data were tested with two-way ANOVA between activated and nonactivated, with Sidak’s multiple comparisons test. All tests were performed with Prism 8 (GraphPad) software.

## Figures and Tables

**Table 1:** Metabolite concentrations for control (blue), M1-activated (red), and M2-activated (orange) J774a.1 macrophages, calculated from high resolution 800 MHz ^1^H NMR spectra of J774a.1 macrophage extracts. Concentration is reported as mean ± standard deviation. N.B.: Metabolites with any significance between groups are underlined and italicized, with *p*-values for significant differences shown in Figure 5B.

J774a.1 Non-Activated vs. Activated Metabolite Concentrations (µM)			
Metabolite	Control	M1-Activated	M2-Activated
Acetate	27 ± 12.7	26.52 ± 11.1	15.18 ± 6.58
Alanine	54.91 ± 24.3	36.48 ± 9.34	43.02 ± 7.98
*Arginine*	20.37 ± 7.68	26.17 ± 7.24	14.28 ± 4.75
*Aspartate*	76.93 ± 36.1	21.05 ± 20.89	50.57 ± 10.53
*Choline*	5.86 ± 2.23	13.13 ± 12.5	2.58 ± 0.72
Citrate	21.16 ± 10.57	10.03 ± 2.69	11.76 ± 2.38
*Creatine*	16.72 ± 7.54	22.59 ± 4.31	13.69 ± 3.45
Glutamate	111.09 ± 54.14	49.98 ± 6.89	69.95 ± 14.23
*Glutamine*	36.97 ± 14.99	78.15 ± 31.56	24.51 ± 7.28
Glutathione	48.79 ± 19.16	68.46 ± 26.94	46.3 ± 17.43
*Itaconic acid*	2.13 ± 1.74	40.27 ± 4.59	0.74 ± 0.71
*Lactate*	406.03 ± 83.92	748.64 ± 310.05	280.91 ± 49.32
*NAD*	6.2 ± 1.45	3.93 ± 1.09	2.94 ± 0.45
*Succinate*	4.93 ± 5.81	19.86 ± 1.4	3.38 ± 1.34
*Taurine*	90.85 ± 21.66	244.7 ± 92.42	113.2 ± 49.72

## Data Availability

The data presented in this study are available on request from the corresponding author.

## Supplementary Materials

The following are available online at https://www.mdpi.com/article/10.3390/metabo11070410/s1, Figure S1: M1 and M2 activation of J774a.1 macrophages.
