# Supplementary material for: Non-Invasive Differentiation of M1 and M2 Activation in Macrophages Using Hyperpolarized 13C MRS of Pyruvate and DHA at 1.47 Tesla
Source: Metabolites. 2021 Jun 22;11(7):410. doi: 10.3390/metabo11070410 (PMC8305442; doi:10.3390/metabo11070410)
Supplement: Supplementary file 1 [file metabolites-11-00410-s001.zip › metabolites-1213160-supplementary.pdf]

Supplementary Figures

Supplementary Figure S1

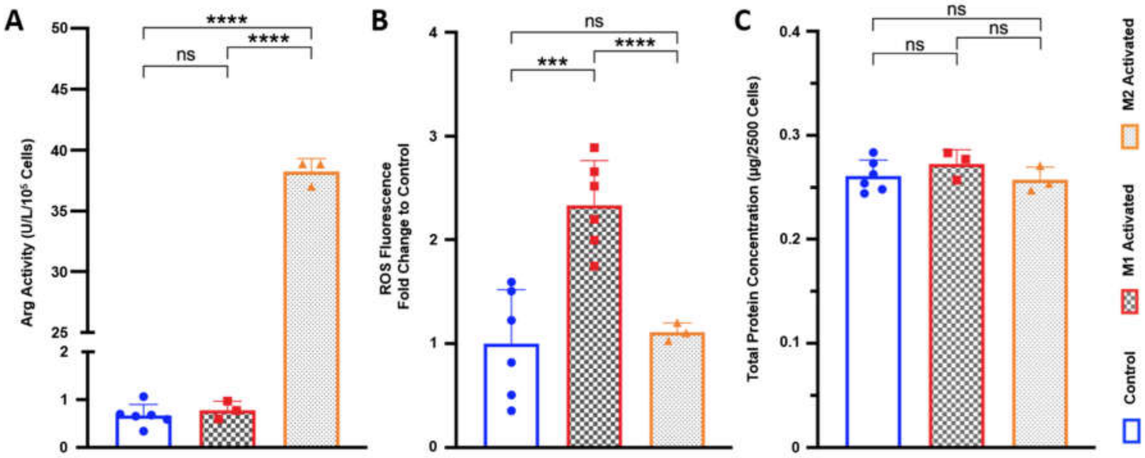

Supplementary Figure S1. M1 and M2 activation of J774a.1 macrophages. (**A**) Arginase (Arg) activity in Units (where 1 Unit = amount of arginase that will generate 1.0 nmol of H_2O_2/min at 37°C) for control (blue), M1 activated (red) and M2 activated (orange) macrophages (ns: p = 0.849, ****p<0.0001). (**B**) Reactive oxygen species (ROS) fluorescence levels between control (blue), M1 activated, (red) and M2 activated (orange) J774a.1 macrophages, expressed in fold change compared to control (ns: p = 0.738, ***p<0.001, ****p<0.0001). (**C**) Total protein concentrations normalized to cell numbers for control (blue), M1 activated, (red) and M2 activated (orange) J774a.1 macrophages (ns control vs M1: p = 0.309; ns control vs M2: p = 0.721; ns M1 vs M2: p = 0.229).
